# Longitudinal Cytokine Profiling Identifies GRO-α and EGF as Potential Biomarkers of Disease Progression in Essential Thrombocythemia

**DOI:** 10.1097/HS9.0000000000000371

**Published:** 2020-05-21

**Authors:** Nina F. Øbro, Jacob Grinfeld, Miriam Belmonte, Melissa Irvine, Mairi S. Shepherd, Tata Nageswara Rao, Axel Karow, Lisa M. Riedel, Oliva B. Harris, E. Joanna Baxter, Jyoti Nangalia, Anna Godfrey, Claire N. Harrison, Juan Li, Radek C. Skoda, Peter J. Campbell, Anthony R. Green, David G. Kent

**Affiliations:** 1 Wellcome MRC Cambridge Stem Cell Institute, University of Cambridge, Hills Road, Cambridge, CB2 0XY, United Kingdom; 2 Department of Hematology, University of Cambridge, CB2 0XY, United Kingdom; 3 Department of Hematology, Cambridge University Hospitals NHS Foundation Trust, Hills Road, Cambridge CB2 0QQ, United Kingdom; 4 York Biomedical Research Institute, Department of Biology, University of York, York, YO10 5NG, United Kingdom; 5 Experimental Hematology, Department of Biomedicine, University Hospital Basel and University of Basel, 4031 Basel, Switzerland; 6 Department of Pediatrics and Adolescent Medicine, Friedrich-Alexander-Universität Erlangen-Nürnberg (FAU), Erlangen, Germany; 7 Wellcome Trust Sanger Institute, Wellcome Genome Campus, Hinxton, United Kingdom; 8 Department of Hematology, Guy's and St. Thomas’ NHS Foundation Trust, London, United Kingdom

## Abstract

Myeloproliferative neoplasms (MPNs) are characterized by deregulation of mature blood cell production and increased risk of myelofibrosis (MF) and leukemic transformation. Numerous driver mutations have been identified but substantial disease heterogeneity remains unexplained, implying the involvement of additional as yet unidentified factors. The inflammatory microenvironment has recently attracted attention as a crucial factor in MPN biology, in particular whether inflammatory cytokines and chemokines contribute to disease establishment or progression. Here we present a large-scale study of serum cytokine profiles in more than 400 MPN patients and identify an essential thrombocythemia (ET)-specific inflammatory cytokine signature consisting of Eotaxin, GRO-α, and EGF. Levels of 2 of these markers (GRO-α and EGF) in ET patients were associated with disease transformation in initial sample collection (GRO-α) or longitudinal sampling (EGF). In ET patients with extensive genomic profiling data (n = 183) cytokine levels added significant prognostic value for predicting transformation from ET to MF. Furthermore, CD56^+^CD14^+^ pro-inflammatory monocytes were identified as a novel source of increased GRO-α levels. These data implicate the immune cell microenvironment as a significant player in ET disease evolution and illustrate the utility of cytokines as potential biomarkers for reaching beyond genomic classification for disease stratification and monitoring.

## Introduction

Leukemia is typically associated with the step-wise acquisition of mutations, and large-scale genomic profiling efforts have identified the major driver mutations in hematological malignancies.^1,2^ Despite these efforts, significant disease heterogeneity persists across patients with identical driver mutation profiles. Comparatively little is known about the cellular environment in which these cells reside and what impact neighboring cells, or the molecules they secrete, might have on disease evolution. A powerful disease model for studying the early stages of tumorigenesis is the chronic myeloproliferative neoplasms (MPNs), where a fraction of patients progresses to either develop bone marrow fibrosis or, in the most severe cases, transform to acute leukemia.

Key studies emerging from the field of pre-leukemic diseases and leukemia suggest that the inflammatory microenvironment plays a significant role in disease establishment and maintenance.^3–5^ Recent clinical and biological findings point to a role for chronic inflammation as a key extrinsic factor, driving aspects of Philadelphia Chromosome negative myeloproliferative neoplasm (MPN) pathogenesis and progression.^6–9^

The role that cytokines play as regulators of normal hematopoiesis is widely recognized, and the balance between self-renewal and differentiation in hematopoietic stem cells (HSCs) is tightly regulated by micro-environmental factors both in steady-state and during stress.^10^ Deregulation of inflammatory cytokines in MPN patients has been reported in a number of previous studies^11–17^ and IL-8 and IL2R levels were associated with overall survival in the most severe MPN subtype myelofibrosis (MF).^12^ However, the vast majority of studies did not profile large numbers of the less severe subtype essential thrombocythemia (ET) patients and samples were often from a single static timepoint.

Here, we undertake a comprehensive serum cytokine profile of more than 400 MPN patient samples. Cytokine data and longitudinal sampling were integrated with clinical and genetic information to determine whether potential biomarkers for adverse prognosis or disease monitoring could be identified. Levels of 2 such markers, GRO-α and EGF, were identified as indicators of transformation-free survival in pre-treatment and longitudinal samples respectively, adding significant prognostic information beyond genetic profiling.

## Methods

### Patient samples

The study included three groups of Philadelphia chromosome negative MPN patients: (A) Cambridge cohort (n = 291 patients), (B) Swiss cohort (n = 204 patients), and (C) a selected PT-1 cohort (n = 122 patients) enriched for MF and AML transformation events (Supplemental Appendix 1). Patients from UK cohorts (A and C) were diagnosed according to the British Committee for Standards in Hematology (BCSH) guidelines.^18^ Diagnosis in the Swiss cohort was established according to the revised criteria of the World Health Organization.^19^ UK patient samples were obtained under local ethics approval (Cambridge and Eastern Region Ethics Committee), or as part of the UK Medical Research Council PT-1 trial.^20–23^ For the Swiss cohort, samples and clinical data were obtained at the study center in Basel (Switzerland), and approved by the local ethics committees (Ethik Kommission Beider Basel). Written informed consent was obtained from all patients in accordance with the Declaration of Helsinki. For local Cambridge patients, samples were taken at the time of the initial patient visit on referral to the specialist MPN clinic. For the PT-1 patient cohort, 36% of patients had their samples collected before receiving any cytoreductive therapy and >80% of patients were sampled within the first 30 days (Supplemental Appendix 1). From the UK patient cohorts, targeted sequencing of the coding regions of 33 recurrently mutated genes was available for 239 patients (n = 117 from Cambridge cohort, n = 122 from PT1 cohort).^24^ Longitudinal studies included repeat samples from UK patient cohorts (n = 81). Cytoreductive treatment information was available in PT1 cohort patients and included in multivariate analyses. Baseline bone marrow fibrosis data was available for a subset of patients (n = 44).

### Patient serum cytokine profiling

Blood was collected in Becton Dickenson (BD) Vacutainer tubes or S-Monovette Z Gel clot activator tubes (Sarstedt). After clot formation and centrifugation, serum aliquots were stored at − 80°C or in liquid nitrogen (Cambridge Blood Stem Cell Biobank). An initial 38-plex panel (Milliplex HCYTOMAG-60K-PX38) was run on 185 MPN patients and 14 healthy controls. A 10-plex custom assay (IP-10, IL-8, EGF, eotaxin, TGF-α, IFN-γ, GRO-α, IL1-RA, TNF-α, IL-6) was designed to profile a further 106 MPN cases (total n = 291), as well as an additional 122 ET patients for biomarker validation (PT-1 cohort). Each immunoassay was completed using 25 μL serum (undiluted) and was performed according to the manufacturer's protocol, and protein quantification was undertaken to ensure that cytokine levels were not skewed by overall protein content (Supplementary Table 1). Plates were run on Luminex xMAP (Luminex Corp., Austin, TX) and cytokine concentrations were determined by xPONENT software (Luminex), using values derived from the known reference concentrations. Serum cytokine profiling (25 cytokines) in the Swiss cohort was performed using the Meso Scale Discovery Platform (Rockville, MD) according to the manufacturer's instructions.

### Intracellular flow cytometry

Fresh peripheral blood samples were collected in Lithium Heparin tubes (Addenbrooke's MPN Clinic), and mononuclear cells (MNCs) were isolated by density gradient centrifugation (Lymphoprep, Axis-Shield, Oslo, Norway). MNCs were plated at 2–5 × 10^6^ cells/mL in RPMI (Sigma) supplemented with 10% fetal calf serum plus secretion inhibitor (GolgiSTOP, BD Biosciences (BD), 0.6 μL/well) and were stimulated for 4 hours with 100 ng/mL lipopolysaccharide (LPS, Sigma). After stimulation, cells were washed, treated with Fc-Receptor Blocking (BD Biosciences) and stained for cell surface markers CD45/V450 (HI30, BD), CD3/APC, CD14/PE-Cy7 (M5E2, BD or BioLegend), CD15 BV605 (W6D3, BD), CD56/BV711 (NCAM16.2, BD) and Zombie Aqua (Biolegend) for live/dead cell discrimination. Cells were washed, fixed and permeabilized (fixation/permeabilization solution, BD), washed in perm/wash buffer (BD) before intracellular stain using GRO-α/PE (#20326, R&D). Samples were run on a BD Fortessa and analyzed in FlowJo10. After gating for CD45^+^ live singlets (using FSC-A, SSC-A, FSC-H), total GRO-α^+^ cells were gated as percentage of total live MNCs using the unstimulated level in healthy control samples as threshold for positivity. The following MNC subsets were gated on mature cell markers: Monocytes (CD14^+^CD15^−^), T-cells (CD3^+^CD56^−^CD14^-^CD15^−^CD19^−^), NK-cells (CD3^−^CD56^+^CD14^−^ CD15^−^CD19^−^), and NKT-cells (CD3^+^CD56^+^CD14^−^CD15^−^CD19^−^) (Supplemental Fig. 3). The proportion of MNC subsets producing GRO-α was analyzed as the percentages of monocytes (CD56^−^ and CD56^+^ subsets), T-, NK-, and NKT-cells of total GRO-α^+^ cells.

### Statistical analysis

Kruskal-Wallis and Mann-Whitney *U* tests (GraphPad Prism7 and R) were used for comparison of cytokine concentrations between patient subgroups. Fisher Exact test was used for proportional MNC subtype analyses of GRO-α flow cytometry data. Kaplan-Meier and Cox proportional hazards modeling (for time-to-event analyses), random forest, and mixed effects modeling were done in R (v3.2.2). The R packages used were ggplot2 (v2.2.1), randomForest (v4.6-12), RMS (vs5.1-0), survival (v2.40-1) and lme4 (v1.1-12). Multivariate analyses included: age at diagnosis, sex, white cell and platelet counts, and where available and specifically stated in sub-group analyses: mutation status (0/1) for recurrently mutated genes (JAK2, CALR, MPL, TET2, DNMT3A, ASXL1, SRSF2, U2AF1, IDH2, SF3B1, and CBL), reticulin grade at diagnosis and length of treatment on anagrelide or hydroxycarbamide. These 11 genes all occurred in >2% of patients and have been previously implicated in MPN pathogenesis. A spreadsheet of all patient data analyzed can be found as Supplemental Appendix 1.

## Results

### MPNs have disease-specific patterns of inflammatory molecules

**Figure 1:**
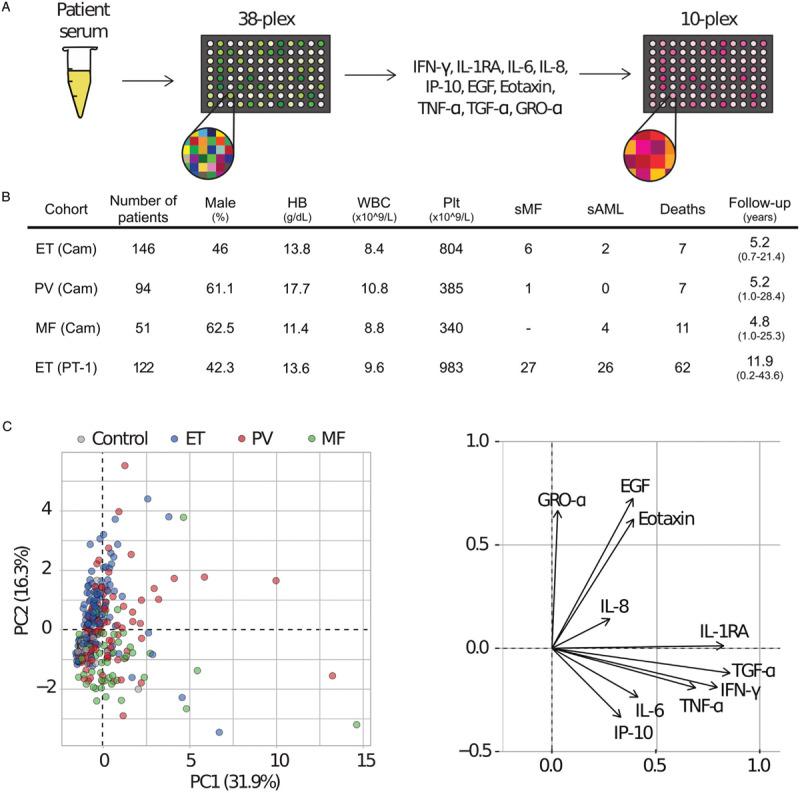
**Serum cytokine profiling identifies distinct cytokine networks in MPNs**. (A) Overview of serum cytokine screen. Initially levels of 38 cytokines were assessed in serum samples from MPN patients by Luminex-based multiplexed ELISA. Following data analysis, 10 cytokines were selected for their ability to track with disease subtypes, disease severity, or overall survival. (B) Summary information for patient groups. Median follow-up time is displayed in years with minimum/maximum follow-up indicated in parenthesis. Median values are displayed for Hb, WBC, and Plt. (C) Principal components analysis plot displaying the serum samples from 291 MPN patients (PC1 = 31.9%, PC2 = 16.3%) with ET (blue circles) and MF (green circles) patient samples positioned in distinct areas. Data are from the initial Cambridge cohort where all disease subtypes were collected in an unbiased fashion (ET n = 146, PV n = 94, PMF n = 51) and normal controls (n = 14). The right panel shows the loadings plot identifying cytokines accounting for the differences. ET = essential thrombocythemia, PV = polycythemia vera, MF = primary myelofibrosis, Hb = hemoglobin, WBC = white blood cell count, Plt = platelet count, sMF = secondary MF, sAML = secondary AML.

Thirty-eight inflammatory molecules were quantified in serum from an initial 185 patients from the Cambridge cohort (104 ET, 52 polycythemia vera (PV) and 29 primary myelofibrosis hereafter referred to as MF (Fig. 1A-B), to identify those that were most discriminatory for MPN subtype (Supplemental Fig. 1A). When disease subtypes were considered as single entities against each other (and normal controls), a number of cytokines were significantly altered and showed strong disease specificity. Each of EGF, eotaxin, GRO-α, TGF-α, IL-1RA, TNF-α, IL-6, IL-8, IP-10, and IFN-γ, were significantly different for at least one MPN subtype (Supplemental Fig. 1B) or associated with decreased overall survival (IL-6, p = 0.01). These informative cytokines were combined in a custom 10-plex array used for all further studies.

To further investigate these findings, we extended the Cambridge cohort to a total of 291 patient patients (146 ET, 94 PV, 51 PMF) (Fig. 1 A-B) for analyses using the custom 10-plex array. Group comparison for the individual cytokines and principal component analysis of the full dataset further confirmed distinct inflammatory cytokine networks associated with the ET, PV or PMF subtypes (Fig. 1C). PMF was associated with increased levels of TNF-α, IP-10 and IL-8 (Supplemental Figure 2A), which is consistent with previous studies^12^ and these findings were reinforced by results from a separate Swiss cohort (n = 204 patients, n = 24 controls) analyzed with a different screening platform (Supplemental Fig. 2B).

Comprehensive data on mutation status for genes commonly mutated in MPNs, as well as recurrent chromosomal abnormalities (including JAK2, CALR, MPL, TET2, DNMT3A, ASXL1, EZH2, SRSF2, 9pUPD, and del(20q)), was available for a majority of patients.^24^ After correction for diagnosis, age and sex, surprisingly few differences in cytokine levels were observed across patients based on mutational status alone, suggesting that micro-environmental heterogeneity was not dominantly instructed by a particular genetic lesion or combination of lesions. The single exception was IP-10, which showed positive correlation with JAK2V617F variant allele fraction in ET (p = 0.023), PV (p = 0.0061), and MF (p = 0.027) patients.

**Figure 2:**
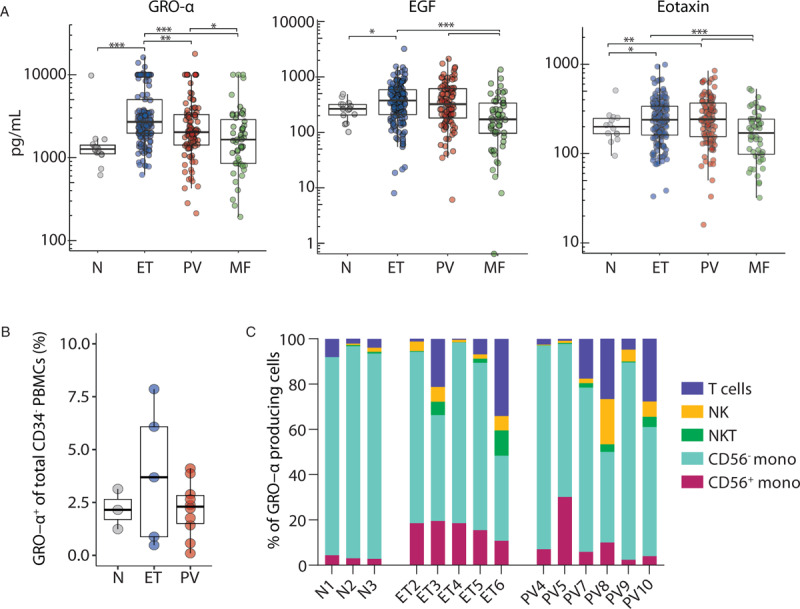
**Elevated levels of EGF, eotaxin, and GRO-α in patients with essential thrombocythemia**. (A) Serum levels of individual cytokines that are increased in patients with essential thrombocythemia compared to other MPN subtypes. Initial Cambridge cohort (ET n = 146, PV n = 94, MF (primary myelofibrosis) n = 51) and normal controls (n = 14). Boxes show medians with interquartile range (IQR). Mann-Whitney *U* test ^∗^p < 0.05, ^∗∗^p < 0.01, ^∗∗∗^p < 0.001, ^∗∗∗∗^p < 0.0001. (B) Peripheral blood MNCs from MPN patients were stimulated with LPS for 4 hours and GRO-α production measured by flow cytometry (% positive of total MNCs) (Normal controls n = 3, ET = 5, PV = 10). (C) Cell types producing GRO-α after LPS stimulation was evaluated using surface markers for T-cells, NK-cells, NKT-cells, CD56^−^ monocytes, and CD56^+^ monocytes (Normal controls n = 3, ET n = 5, PV n = 6). The proportion of cellular sources of GRO-α in patients are displayed in a stacked bar graph, showing a higher frequency of GRO-α^+^CD56^+^CD14^+^ monocytes observed in ET patients compared to normal controls (p = 0.008).

Previous studies have concluded that relatively lower levels of pro-inflammatory cytokines/chemokines are present in patients with ET compared to those with PV and MF patients^8^, although these studies had very few ET patients (n = 5, n = 15, and n = 21)^13,14,16^ compared to our cohort of 146 ET patients, and/or studied a smaller panel of cytokines. Counter to this notion, we found that GRO-α (CXCL1) levels were markedly raised in ET patients compared to other MPNs, while EGF and eotaxin (CCL11) were higher in both ET and PV compared to MF patients (Fig. 2A). These findings were confirmed by multivariate analysis (ANOVA) that included age and sex.

### CD56^+^CD14^+^ pro-inflammatory monocytes drive over-production of GRO-α

To further examine the different disease-specific microenvironments, we next surveyed various mature blood cell types (T-, NK-, NKT-cells, and monocytes) by intra-cellular flow cytometry to determine the cellular origin of GRO-α production in patients (Supplemental Fig. 3). Following LPS stimulation, GRO-α production in total PB MNCs ranged from 1% to 8% (Fig. 2B) with a proportion of ET patients having substantially higher numbers of GRO-α producing cells. Monocytes were the predominant producer of GRO-α in all patient samples, with the proportion of GRO-α^+^CD56^+^CD14^+^ monocytes being significantly increased in ET patient samples compared to normal controls (p = 0.008, Fig. 2C). CD56^+^CD14^+^ monocytes are known to be a pro-inflammatory subset of monocytes,^25^ increased levels of which have been found in patients with solid tumors and hematological malignancies.^26^ These data suggest a role for CD56^+^CD14^+^ monocytes in creating a high GRO-α environment which may in turn be a driver of MPN disease evolution. Hyperactive monocytes have also been implicated in MF patients, where increased constitutive production of cytokines included TNF-α, IL-10 and TGF-ß.^27^

### GRO-α levels are predictive of transformation in ET patients

**Figure 3:**
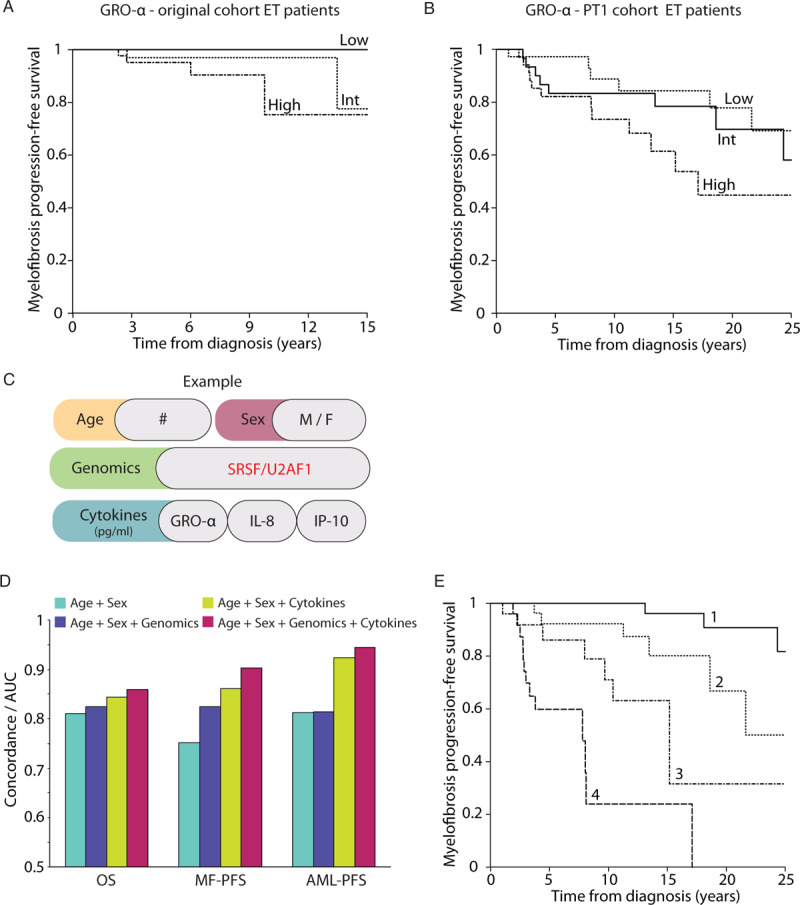
**Cytokine measurements add prognostic value beyond genomics data alone**. (A) Kaplan-Meier analysis of progression-free survival according to pre-transformation levels of GRO-α in ET and PV patients with transformation follow-up data in initial Cam cohort (n = 151). High levels of GRO-α correlate with increased risk of transformation from chronic phase to secondary MF (Cox proportional hazards modeling including age, sex and diagnosis. p = 0.004). B) Kaplan-Meier analysis of progression-free survival in PT-1 cohort. High levels of GRO-α correlate with increased risk of transformation from ET to secondary MF (Cox proportional hazards modeling including age and sex. p = 0.01). (C) Variables considered in the prognostic model included age, sex, levels of 10 cytokines, and presence/absence of 11 driver mutations. (D) The predictive yield (as assessed by model concordance, equivalent to the area under the curve for the receiver-operator characteristic) of adding cytokine quantification to prognostic models utilizing demographic and clinical data alone, and those additionally incorporating genomic variables. Genomic characterization improves the prediction for disease transformation, and inclusion of cytokine measurements further improves predictive power. OS = overall survival; MF-PFS = myelofibrosis progression-free survival; and AML-PFS = AML progression-free survival with transformation follow-up data (n = 122). E**)** Kaplan-Meier curve of progression-free survival where patients have been stratified into equally sized groups (quartiles of 30 patients each) according to their predicted risk as defined by a multivariate Cox proportional hazards model using age, GRO-α levels, IL-8 levels, IP-10 levels, and presence/absence of an SRSF2/U2AF1 mutation (p < 0.001).

Comprehensive long-term clinical outcome data was available for 182 of the 291 patients in the original Cambridge cohort and these data were used to determine whether cytokine levels might be useful as predictive prognostic biomarkers. Of these, data in ET patients (n = 116), showed that high levels of GRO-α correlated with an increased risk of transformation from ET to MF (Cox proportional hazards modeling, p = 0.004) (Fig. 3A). However, since this ET cohort only had 8 transformation events (6 secondary MF (sMF), 2 sAML), we extended our 10-plex serum cytokine analysis to a further 122 ET patients from the PT1 study.^20–22^ From the >1200 patients in the PT-1 trial, we selected a cohort that was highly enriched for patients with transformation events (26 sAMLs, 30 sMFs, 69 deaths) to directly address the question of whether initial GRO-α levels could predict transformation. The PT1 cohort had genomic and clinical data was available with a median of 11.7 years of follow-up (range 2 months to 43 years from diagnosis). In this dataset, the correlation of high GRO-α levels with risk of ET to sMF transformation was confirmed (p = 0.01, Fig. 3B), although GRO-α levels did not associate with sAML transformation (data not shown). ‘Baseline BM fibrosis grade, available for 44 patients, showed no significant association with GRO-α level (p = 0.83), and the predictive value of GRO-α remained significant after inclusion of fibrosis grade as a covariate in this set of patients (p = 0.005). Fourteen patients with low or intermediate GRO-α level together with reticulin grade 0–1 had no MF transformation, compared with transformation occurring in half of the remaining 30 patients with either high GRO-α or fibrosis grade 2–3 at baseline. Notably, diagnostic PMF samples did not have elevated GRO-α levels, suggesting that high levels are predictive of future chronic phase transformation rather than a hallmark feature of myelofibrosis.

### Cytokine profiling improves on genomics for predicting transformation-free survival

We next assessed adding cytokine quantification to prognostic models utilizing demographic and clinical data alone, and those additionally incorporating genomic variables (Fig. 3C). Here, we assessed age, sex, levels of all 10 cytokines and the presence or absence of 33 genetic mutations for their capacity to predict transformation to sMF or sAML and survival in MPN patients. First, this analysis confirmed a recently published study showing that comprehensive genomic characterization can improve the prediction for disease transformation.^24^ Second, inclusion of cytokine levels improved the performance of the prognostic model, as assessed by the Concordance-statistic for goodness of fit (Fig. 3D). Stepwise variable selection identified a prognostic model that included age, presence/absence of U2AF1 or SRSF2 mutations, GRO-α, IL-8 (p <0.01) and IP-10 (p = 0.12) levels (Fig.3E).

### Longitudinal monitoring of EGF levels predicts transformation events

**Figure 4:**
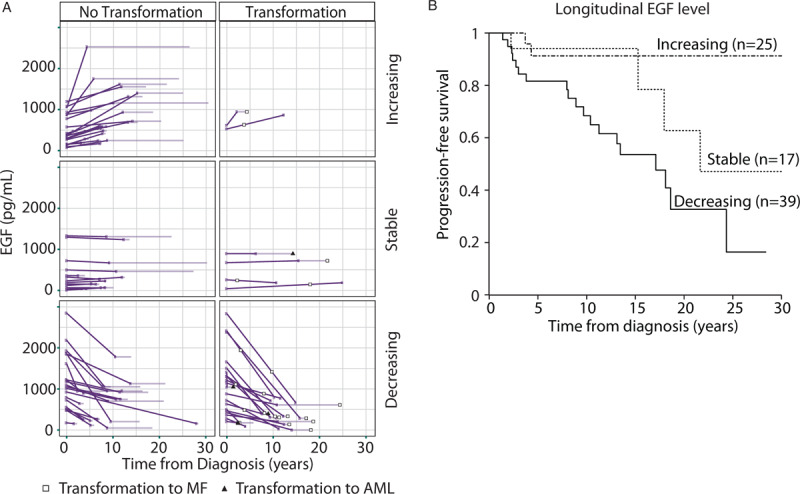
**Decreases in EGF levels over time associate with MF transformation**. (A) EGF levels were assessed at multiple time points during disease and patients were classified as having increasing, decreasing, or stable levels of EGF. EGF stability was defined as an absolute rate of change of <8pg/mL per year. The 46% (18/39) of patients with decreased levels of EGF over time transformed to MF or AML, while only 24% (4/17) and 8% (2/25) of patients with stable and increasing levels of EGF respectively transformed to MF or AML. Dark lines indicate the point up until which sampling occurred and light lines indicate non-sampled follow-up. Longitudinal samples were taken pre- or post-transformation and are separated accordingly. (B) MF progression-free survival of patient cohorts defined by increasing, stable or decreasing EGF levels over time. Patients in whom levels of EGF decreased were 4.3-fold (95% confidence interval 1.7–10.9) more likely to transform to MF or AML (p value = 0.008).

We extended our study to longitudinal samples (n = 81) to determine whether any of the chronic phase markers (EGF, eotaxin, and GRO-α) might be useful for monitoring disease for risk of subsequent transformation. Here it was observed that the rate of change in EGF levels strongly correlated with transformation risk (p = 0.008), with the vast majority of transformations (∼80%) observed in patients whose EGF levels decreased over time (Fig. 4A and B), where stability was defined as an absolute rate of change of <8pg/mL per year. Compared to patients with stable or increasing EGF levels, there was a 4.3-fold (95% confidence interval 1.7–10.9) increased likelihood of transformation to sMF or sAML when EGF levels dropped over the course of the longitudinal sampling. The association between decreasing EGF levels and risk of myelofibrotic transformation remained significant after correction for baseline reticulin grade and treatment with anagrelide or hydroxycarbamide (p = 0.02). This indicates the potential utility of monitoring EGF levels during the course of disease adding a dynamic measurement of disease evolution which would reach beyond traditional binary assessments of mutant/non-mutant that are common with genomic profiling.

## Discussion

Recent studies have explored the pivotal role for the microenvironment in the establishment and maintenance of myeloid malignancies.^3,28–30^ High levels of inflammatory cytokines have been observed in myelofibrosis,^11–13^ MDS,^31,32^ and AML^32^ and IFN-α is an established treatment in the management of myeloid malignancies.^33^ In this study, serum cytokine profiling spanning MPN subtypes identified 10 cytokines that associate with disease subtype and/or disease severity. Moreover, prognostic modeling demonstrated that measuring cytokines could complement clinical and genomic information for the prediction of transformation-free survival, suggesting they may be useful for disease monitoring and assessment.

Our data identify a small collection of inflammatory cytokines (GRO-α, EGF, and eotaxin) that are elevated in chronic phase MPNs. Previous ET patient data concluded that relatively low levels of chemokines, pro- or anti-inflammatory cytokines, or hematopoietic growth factors were present in patients with ET compared to those with PV and MF.^8^ However, these studies often had very few patients (n = 5 in Ho et al,^14^ n = 21 in Pourcelot et al,^16^ n = 15 in Boissinot et al^13^) and only one of these studies measured GRO-α, EGF, or eotaxin levels^14^ with no significant changes observed, likely due to the small number of ET patients sampled (n = 5). Our study of all MPN subtypes comprising 146 ET patients, was the first to be sufficiently powered to identify ET-specific differences in GRO-α, EGF, and eotaxin levels and clearly demonstrates their dysregulation in ET patients.

Surveying genetic mutations in myeloid malignancies has become increasingly utilized for predicting outcomes,^24,34^ but mutations cannot fully explain disease heterogeneity. This could be due to different stages of disease evolution, a different cell of origin,^35^ different sized mutant clones,^36^ different orders of mutation acquisition,^37^ or other factors not yet tracked. Here we demonstrate that cytokine measurements have substantial prognostic value for predicting transformation in addition to age, demographics and comprehensive genomics. Specifically, single time-point cytokine measurements demonstrated predictive utility, with high levels of GRO-α being predictive of MF transformation in both the initial and validation ET patient cohorts, and decreasing EGF levels over time predicting for transformation in longitudinal sampling. Of particular interest, measuring longitudinal changes in EGF levels goes beyond the traditional binary assessment of mutant/non-mutant status that is often done with genomic profiling, although it would require a prospective trial with MPN and healthy controls that has set analysis timepoints and tests the utility of an 8pg/mL/year rate of change.

Altered cytokine levels in MPN patients might derive from hematopoietic (clonal and/or non-clonal) or non-hematopoietic cell sources.^38^ One potential mechanism is the clonal expansion of megakaryocytes and monocytes associated with MPNs. Increases in cell number might translate into higher levels of specific cytokines, potentially driving increased angiogenesis or fibroblast differentiation/recruitment with consequent bone marrow fibrosis.^39,40^ GRO-α has been associated with high platelet counts^41^ and has been previously implicated in vascular disease^42,43^ and we identify CD56^+^CD14^+^ monocytes as a potential source of GRO-α. Also of interest, other groups have demonstrated that loss-of-function TET2 mutations associate with macrophage-mediated inflammation,^44^ cardiovascular disease^45,46^ and co-operate with microbial infections to drive myeloproliferation.^47^ It is also possible that additional treatment agents (immunosuppressants, steroids, etc) could alter cytokine levels. Clearly, the number of variables relevant to disease pathogenesis extends beyond genetic changes in an individual, indicating a strong need to generate comprehensive biological, genetic, and clinical datasets. Such datasets will be essential to define which inflammatory mediators play a dominant role in MPNs and detail how MPN-associated inflammatory cytokines might contribute to disease evolution.

## Acknowledgments

The authors thank Richard Grenfell and Mateusz Strzelecki in the Cancer Research UK Cambridge Institute Flow Cytometry core and Anna Petrunkina-Harrison in the NIHR BRC Cell Phenotyping Hub for technical assistance and suggestions; Helen Jolin, Jillian Barlow and Andrew McKenzie for advice and assistance with the multi-plexed ELISA assays; the Cambridge Blood and Stem Cell Biobank; Patricia Harrington, James Roberts, Hayley Protheroe, and Daniel Adams for clinical sample retrieval, provision and data management; Daniel Bode for help with figure preparation; and Daniel Hodson and Elisa Laurenti for helpful discussion.

## Supplementary Material

Supplemental Digital Content

## Supplementary Material

Supplemental Digital Content
