# Supplementary material for: Longitudinal Cytokine Profiling Identifies GRO-α and EGF as Potential Biomarkers of Disease Progression in Essential Thrombocythemia
Source: Hemasphere. 2020 May 21;4(3):e371. doi: 10.1097/HS9.0000000000000371 (PMC7306314; doi:10.1097/HS9.0000000000000371)
Supplement: Supplemental Digital Content [file hs9-4-e371-s002.pdf]

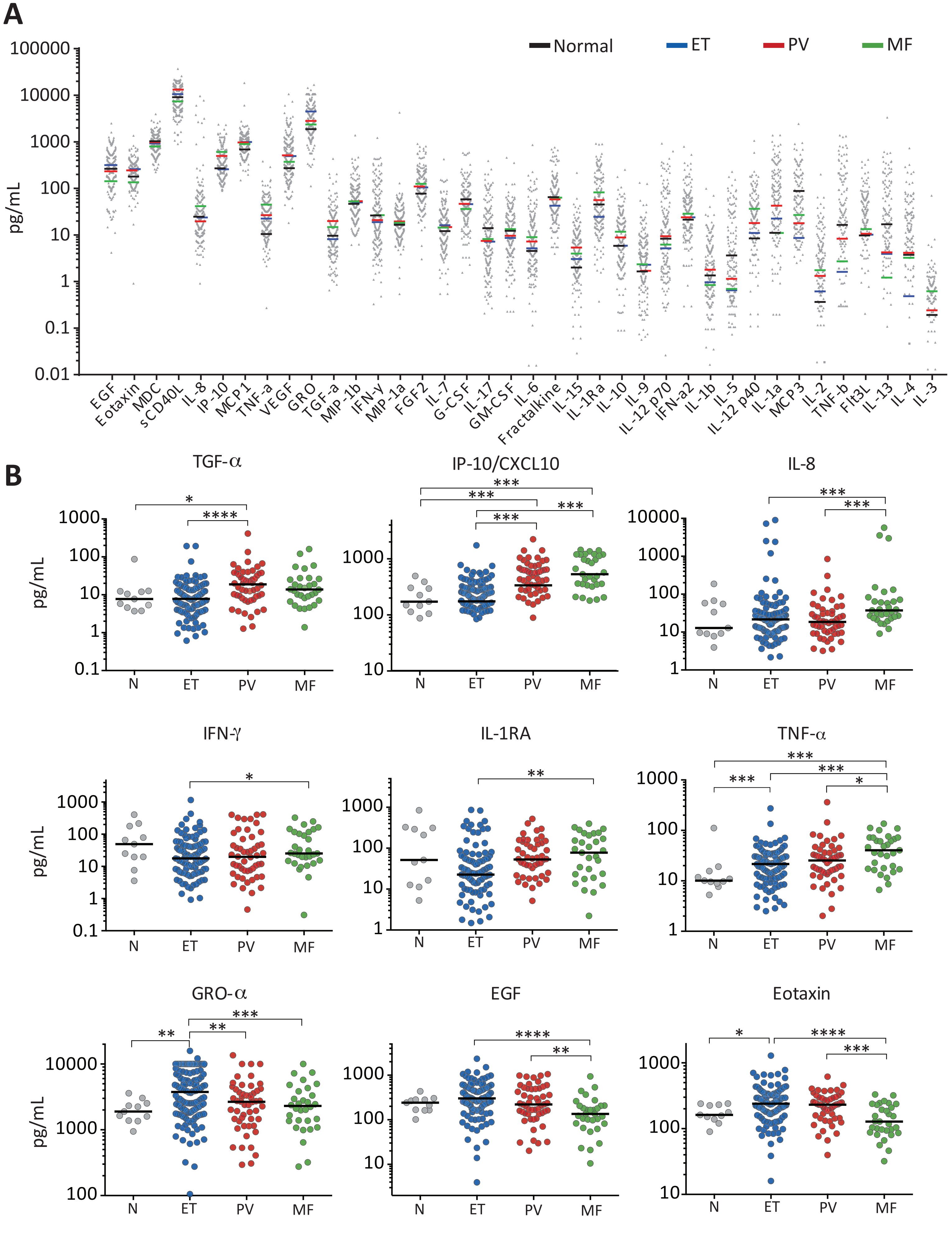
A
Normal
ET
PV
MF
pg/mL
100000
10000
1000
100
10
1
0.1
0.01
EGF
Eotaxin
MDC
sCD40L
IL-8
IP-10
MCP1
TNF-a
VEGF
GRO
TGF-a
MIP-1b
IFN-γ
MIP-1a
FGF2
IL-7
G-CSF
IL-17
GM-CSF
IL-6
Fractalkine
IL-15
IL-1Ra
IL-10
IL-9
IL-12 p70
IFN-a2
IL-1b
IL-5
IL-12 p40
IL-1a
MCP3
IL-2
TNF-b
Flt3L
IL-13
IL-4
IL-3
B
TGF-α
IP-10/CXCL10
IL-8
IFN-γ
IL-1RA
TNF-α
GRO-α
EGF
Eotaxin
N
ET
PV
MF

S1

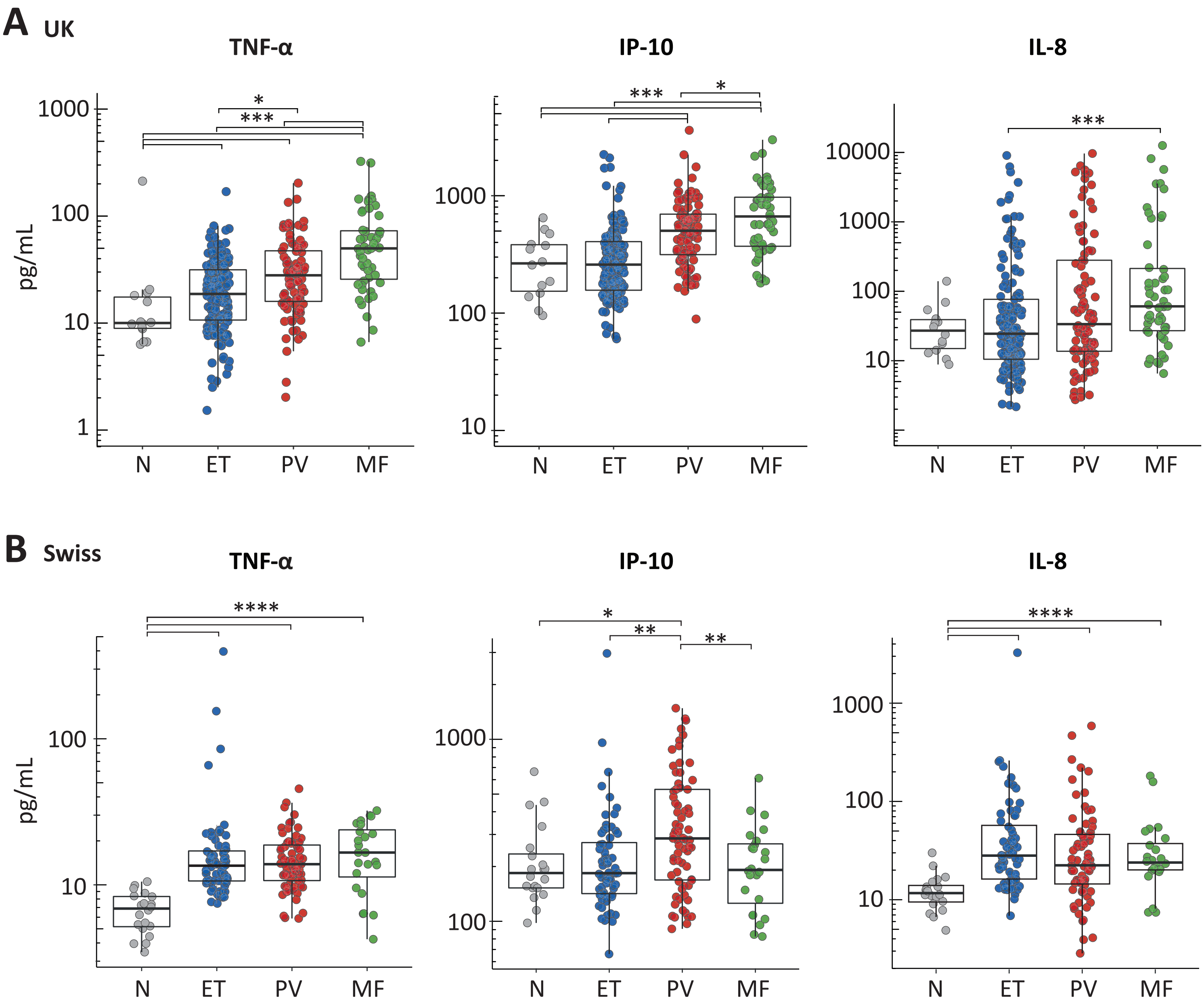

S2

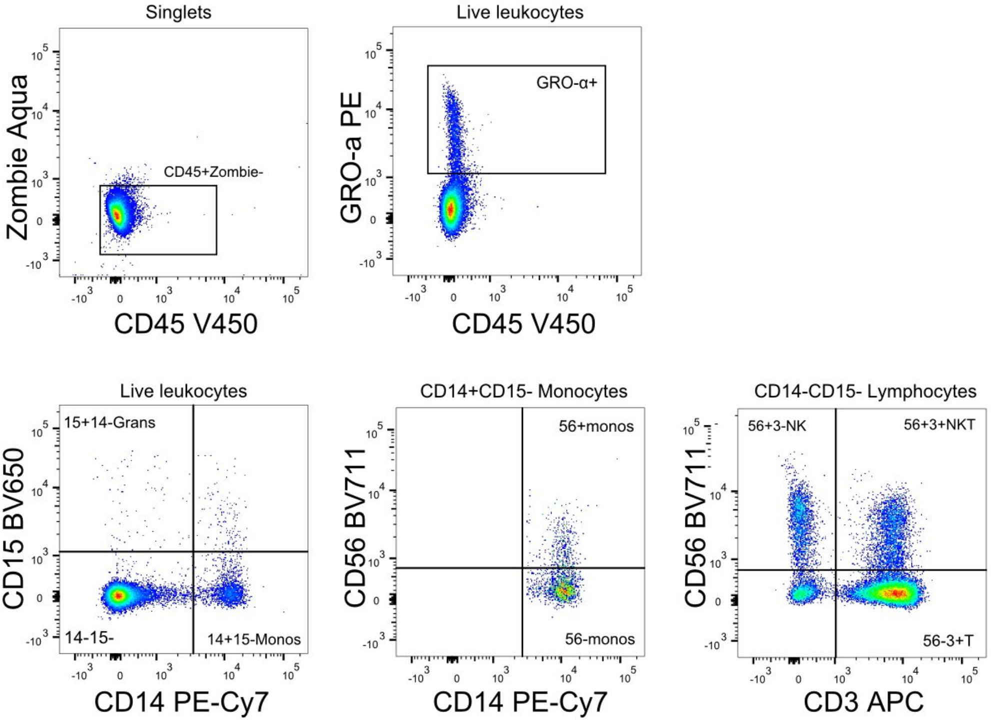
Singlets
Zombie Aqua
CD45+Zombie-
CD45 V450
Live leukocytes
GRO-a PE
GRO-α+
CD45 V450
Live leukocytes
CD15 BV650
15+14-Grans
14-15-
14+15-Monos
CD14 PE-Cy7
CD14+CD15- Monocytes
CD56 BV711
56+monos
56-monos
CD14 PE-Cy7
CD14-CD15- Lymphocytes
CD56 BV711
56+3-NK
56+3+NKT
56-3+T
CD3 APC

S3

Supplemental Figure Legends

Figure S1. 38-cytokine screen of diagnostic serum samples from MPN patients and healthy controls.

A) Serum concentration (pg/mL) of 38 inflammatory cytokines and chemokines across 190 MPN patients and 11 normal individuals. Bars show medians for each MPN subtype (n=11 normal, black line; n=107 ET, blue line; n=52 PV, red line; n=31 MF, green line). **B)** Comparisons of the serum levels of 9 selected cytokines across the three MPN subtypes and normal individuals in the original cohort. Bars show medians. Mann-Whitney U-test *p<0.05, **p<0.01, ***p<0.001, ****p<0.0001.

Figure S2. Serum profile of MF associated cytokines in UK and Swiss MPN cohorts.

A) TNF-α, IP-10, and IL-8 serum levels in 291 MPN patients (146 ET, 94 PV, 51 MF) from the UK cohort. **B)** TNF-α, IP-10, and IL-8 serum levels in 204 MPN patients (ET n=75 PV n=84 MF n=33) and 24 normal controls from Swiss cohort. For the Swiss cohort, samples and clinical data were obtained in Basel, and approved by the local Ethics Committees (Ethik Kommission Beider Basel) and the diagnosis of MPN was established according to the revised World Health Organization criteria. Serum cytokine profiling in the Swiss cohort was performed using Meso Scale Discovery Platform (Rockville, Maryland, USA). Boxes show medians with IQR. Mann-Whitney U-test *p<0.05, **p<0.01, ***p<0.001, ****p<0.0001

Figure S3: Flow cytometry gating strategy.

Data gating strategy used in the intracellular flow cytometry assay to identify GRO-α positive cells and leukocyte subsets CD56+ monocytes, CD56- monocytes, T-cells, NK-cells, and NKT-cells. The plots show a representative example from LPS-stimulated MNCs from a MPN patient.

Correlation between individual cytokines and total protein content

	**Spearman coefficient R**	**p**	**R^2**
TNF-α	-0.11	0.24	0.0121
IP-10	-0.14	0.14	0.0196
IL-8	-0.01	0.91	0.0001
GRO-α	0.16	0.07	0.0256
EGF	-0.04	0.67	0.0015
Eotaxin	0.03	0.73	0.0010
IL-6	-0.08	0.38	0.0066
TGF-α	-0.12	0.20	0.0144
IFN-γ	0.09	0.34	0.0077
IL-1RA	-0.12	0.19	0.0144

Supplementary table 1
